# Pembrolizumab monotherapy in patients with previously treated metastatic high-grade neuroendocrine neoplasms: joint analysis of two prospective, non-randomised trials

**DOI:** 10.1038/s41416-020-0775-0

**Published:** 2020-03-10

**Authors:** Namrata Vijayvergia, Arvind Dasari, Mengying Deng, Samuel Litwin, Taymeyah Al-Toubah, R. Katherine Alpaugh, Efrat Dotan, Michael J. Hall, Nicole M. Ross, Melissa M. Runyen, Crystal S. Denlinger, Daniel M. Halperin, Steven J. Cohen, Paul F. Engstrom, Jonathan R. Strosberg

**Affiliations:** 1 Fox Chase Cancer Center, Philadelphia, PA United States; 2 MD Anderson Cancer Center, Houston, TX United States; 3 H. Lee Moffitt Cancer Center, Tampa, FL United States; 4 Abington Hospital/Jefferson Health, Abington, PA United States

**Keywords:** Neuroendocrine cancer, Phase II trials

## Abstract

**Background:**

Metastatic high-grade neuroendocrine neoplasms (G3NENs) have limited treatment options after progression on platinum-based therapy. We addressed the role of Pembrolizumab in patients with previously treated metastatic G3NENs.

**Methods:**

Two open-label, phase 2 studies enrolled patients with G3NEN (Ki-67 > 20%) to receive Pembrolizumab at 200 mg I.V. every 3 weeks. Radiographic evaluation was conducted every 9 weeks with overall response rate as the primary endpoint.

**Results:**

Between November 2016 and May 2018, 29 patients (13 males/16 females) with G3NENs were enrolled. One patient (3.4%) had an objective response and an additional six patients (20.7%) had stable disease, resulting in a disease control rate of 24.1%. Disease control rate (DCR) at 18 weeks was 10.3% (3/29). There was no difference in the DCR, PFS or OS between the PD-L1-negative and -positive groups (*p* 0.56, 0.88 and 0.55, respectively). Pembrolizumab was well tolerated with only 9 grade 3, and no grade 4 events considered drug-related.

**Conclusions:**

Pembrolizumab can be safely administered to patients with G3NENs but has limited activity as a single agent. Successful completion of our trials suggest studies in G3NENs are feasible and present an unmet need. Further research to identify active combination therapies should be considered.

**Clinical trial registration number:**

NCT02939651 (10/20/2016).

## Background

High-grade (G3) neuroendocrine neoplasms (NENs) account for about 10–20% of malignant extrapulmonary neuroendocrine neoplasms,^1^ and are characterised by a Ki-67 proliferative index >20% and/or mitotic index >20/10 high powered fields (HPF). Historically, WHO classification equated poorly differentiated histology with high tumour grade in NENs; however, an increasing body of literature argues against the notion that these are interchangeable and highlights the heterogeneity of this disease.^2^ Most high-grade NENs are poorly differentiated, aggressive cancers, that are often characterised by exceptionally high proliferative activity (Ki-67 index > 50%) and by somatic mutations in common oncogenes or tumour suppressor genes, such as p53 and Rb1.^3^ A smaller subset consists of well-differentiated high-grade neuroendocrine tumours, which commonly arise in the pancreas, are typically characterised by Ki-67 index ranging from 20–50% and often express mutations in chromatin remodelling genes such as MEN1 and DAXX or ATRX.^4^ Recent WHO NEN classifications recognise the distinction between poorly differentiated neuroendocrine carcinomas (NECs) and well-differentiated, high-grade neuroendocrine tumours (NET G3).^5,6^ Whereas NECs typically respond well to platinum-based chemotherapy, NET G3 tend to be more platinum-resistant, but somewhat less aggressive than NECs.^7–10^

All G3 NENs share a high proclivity for metastatic dissemination even among patients with clinically localised tumours.^11–14^ Recent molecular discoveries have led to therapeutic advances in well-differentiated, low and intermediate-grade NENs with the approval of targeted therapies including everolimus, sunitinib and ^177^Lu-DOTATATE.^15^ In contrast, few therapeutic options are available for G3 NENs, and in the absence of prospective studies, treatment recommendations are often extrapolated from the small cell lung cancer (SCLC) literature.^16^ First-line treatment for NECs typically consists of platinum-based chemotherapy with a modest median progression-free survival (PFS) of 4 months and overall survival (OS) of 11 months.^10,16–19^ There are very little data on treatment outcomes for NET G3: platinum-based treatments are often used despite lower response rates, as well as treatment regimens derived from data in low/intermediate-grade NETs.^10,20^ After first-line therapy, no prospective trials have been conducted and a few small, retrospective studies with chemotherapy (temozolomide, oxaliplatin, taxanes etc.) have dismal outcomes.^9,10,21,22^ The lack of active second line treatment options for G3 NENs highlights the unmet need for drug development in these rare tumour types.

Immune checkpoint blockade is a rapidly advancing therapeutic approach with impressive results in several types of cancers.^23–25^ Immune checkpoint inhibitors (CPI) have shown promising results in SCLC and Merkel Cell Carcinoma, both poorly differentiated NENs with unique biologic characteristics and environmental aetiologies.^26–29^ Recently reported phase 3 randomised trial of carboplatin and etoposide with or without atezolizumab in SCLC, showed an improved median OS in the atezolizumab arm (median OS 12.3 versus 10.3 months, hazard ratio 0.70, 95% CI 0.54–0.91).^30^ The impressive activity of CPIs in these tumours suggests a strong rationale for investigating their role in extrapulmonary G3 NENs.

Additional biologic rationale for evaluating CPI in G3 NENs include their high rate of PD-L1 expression (ranging from 14% to 50% of tumours) and relatively high mutational load, compared to low-intermediate-grade NETs.^31–33^ A high mutational load is thought to increase the chances of immune recognition of neoantigens and may be associated with tumour responsiveness to CPI therapy.^34^ There are limited data on tumour mutation burden in G3 NENs but they frequently harbour multiple mutations in key oncogenic drivers.^33,35^

Two simultaneous studies were undertaken across three academic cancer centres in the United States to assess the efficacy of pembrolizumab, an anti–PD-1 antibody, in patients with advanced, extrapulmonary G3 NENs who had previously received platinum-based therapy. Both studies had similar eligibility criteria (excluded pulmonary neuroendocrine carcinomas) and study designs, which allowed for a joint analysis of the patient level data. GI-087 (referred to as FC hereon) was an investigator sponsored study that recruited 21 patients with G3 NENs at 2 academic centres (Fox Chase Cancer Center, PA and MD Anderson Cancer Center, TX). Moffitt-19207 (referred to as LM hereon) was another investigator sponsored study that recruited eight patients with G3 NENs at the H. Lee Moffitt Cancer Center, FL, and was closed prematurely after results from FC were presented at the annual ASCO meeting 2018. The data presented here are combined results of the two studies.

## Methods

### Patients

Eligible patients were at least 18 years old and had metastatic or unresectable, extrapulmonary G3 NENs (Ki-67 index > 20% with either poorly or well-differentiated histology) that had progressed on at least one line of therapy (FC required prior platinum exposure while LM did not), and had measurable disease according to Response Evaluation Criteria in Solid Tumors (RECIST V1.1); an Eastern Cooperative Oncology Group (ECOG) performance status of 0 or 1; and normal organ and bone marrow function. Key exclusion criteria were any G3 NENs (large or small cell type) of lung origin and Merkel cell carcinoma, a diagnosis of immunodeficiency or ongoing systemic immunosuppressive therapy, active autoimmune disease, concurrent second primary cancer and active central nervous system metastases.

### Study design

Both FC and LM were phase 2, open-label studies. Pembrolizumab, a humanised monoclonal IgG4 antibody (mAb) that blocks PD-1, was administered intravenously at a dose of 200 mg every 3 weeks in both studies. Treatment could continue for a maximum of 2 years or until a complete response, dose-limiting toxic effects, or progressive disease occurred. Patients who appeared to have progression in target or non-target lesions, or to have new lesions were allowed to continue therapy if they were asymptomatic, had an ECOG performance status of 0 or 1, and had no evidence of rapid progression; patients were evaluated 4 weeks later to assess possible further progression. All patients underwent cross-sectional imaging of the chest, abdomen and pelvis at the time of screening and 9 weeks after starting therapy and at 9-week intervals thereafter. Evaluations of scans according to RECIST, version 1.1, were conducted at the institutional level. For all patients in FC, pre-treatment tumour specimens (archived tissue) were obtained when available.

The primary objective of both studies was to determine the clinical efficacy of pembrolizumab in patients with advanced G3 NENs beyond the first-line setting. The primary endpoint was objective response rate (ORR) according to RECIST, version 1.1. Secondary endpoints were PFS and OS. All adverse events were assessed according to NCI Common Terminology Criteria for Adverse Events (CTCAE), version 4.21. Exploratory objectives examined potential laboratory correlates for the clinical activity of pembrolizumab.

### Study oversight and sponsor role

The protocols were approved by the institutional review board at each participating centre, and the studies were conducted in accordance with the Declaration of Helsinki and the International Conference on Harmonization Good Clinical Practice guidelines. All the patients provided written informed consent before study entry. The principal investigators, in collaboration with Merck, were responsible for the design and oversight of the study and the development of the protocol. The manuscript was written and prepared by the authors. All the authors vouch for the accuracy and completeness of the data reported and adherence to the study protocol. Merck Investigator Sponsored Program supported both studies but did not participate in the design of the studies or the collection of data.

### Correlative testing

For patients enrolled into FC, PD-L1 immunohistochemistry (IHC) assay was performed at Qualtek Research Laboratories on formalin-fixed, paraffin-embedded tissue sections using anti–PD-L1 monoclonal antibody clone 22C3 [Merck Research Laboratories]. Tumour sections were also stained with anti-CD8 antibody (clone 144B, Dako) to detect tumour infiltrating lymphocytes (TILs). Appropriate positive and negative controls were included in testing. Samples were scored by board-certified pathologist(s) with documented training. An H&E slide was reviewed for confirmation of tumour presence. PD-L1 staining in the tumour was scored based on reactive tumour cells as well as macrophages and TILs (together referred as mononuclear inflammatory cells) within tumour nests. Macrophages and TILs in tumour induced/associated stroma or the stromal interface were also assessed.

### Statistical consideration

The primary endpoint of both studies was ORR among all treated subjects. Secondary endpoints included PFS (per RECIST 1.1) and OS. The FC study hypothesised that a proportion of patients with favourable response less than 5% would be of no interest. The investigational agent would be of interest if the ORR is at least 20%. Twenty-one patients were needed to test the null hypothesis: *p* < = 0.05 against the alternative hypothesis: *p* > = 0.20 at the 8.5% level of significance and with 82.1% power. If 3 or more patients with favourable response (ORR > / = 14%) were observed, then the null hypothesis was to be rejected.

LM study was developed as a Simon two stage study design with an assumption that a true response of >18% would generate interest in a larger randomised study with a projected enrolment of 15 patients in each stage. Given results of the FC, this study was closed after accrual of eight patients with IRB approval and patient level data merged with the FC database.

PFS and OS were estimated according to the Kaplan-Meier method. Results are presented as an aggregate from both studies and also specifically for the FC cohort where applicable.

## Results

### Patient and tumour characteristics

A total of 29 patients were enrolled. Baseline patient and tumour characteristics are summarised in Table 1. The majority (55%) were female and median age was 56 years (range 27–77 years). 62% of patients had an ECOG PS of 1. Nearly half of tumours (48%) had a Ki-67 index ranging from 20–50%, 41% had Ki-67 > 50% and the Ki-67 index was unknown in 10% (all patients with unknown Ki-67 were NECs with mitotic rate >20 per 10 HPF). Histology was characterised as poorly differentiated in 66 %, well-differentiated in 31% and uncharacterised in 3%. Most patients had gastrointestinal primaries (48%) while 34% originated in the pancreas. One patient with renal and one with thymic primary were included and three had an unknown primary site. Nearly half of patients (45%) had only 1 prior line of therapy. Median duration of time from end of platinum-based therapy to start of study was 14.6 weeks.

**Table 1:** Baseline patient characteristics.

Baseline characteristics	Combined (*N* = 29)	FC (*N* = 21)	LM (*N* = 8)
Age in years median (range)	56 (27–77)	54 (27–73)	58 (27–77)
Gender			
Male *n* (%)	13 (45%)	11 (52%)	2 (25%)
Female *n* (%)	16 (55%)	10 (48%)	6 (75%)
Performance status			
1 *n* (%)	18 (62%)	12 (57%)	
0 *n* (%)	11 (38%)	9 (43%)	2 (25%)
Primary site			
Pancreatic *n* (%)	10 (35%)	6 (29%)	4 (50%)
GI (non-pancreatic) *n* (%)	14 (48%)	13 (62%)	1 (13%)
Non-GI/Unkown n(%)^a^	5^a^ (17%)	2^a^ (9%)	3^a^ (37%)
Ki 67 Score			
≤50% *n* (%)	14 (48%)	10 (47%)	4 (50%)
>50% *n* (%)	12 (42%)	10 (48%)	2 (25%)
Unknown	3 (10%)	1(5%)	2 (25%)
Differentiation^b^			
Well-differentiated	9 (31%)	6 (28%)	3 (37%)
Poorly differentiated	19 (66%)	14 (67%)	5 (63%)
Liver metastatic			
Yes	22 (76%)	15 (71%)	7 (87%)
No	7 (24%)	6 (29%)	1 (13%)
No. of prior therapy			
1 *n* (%)	13 (45%)	8 (38%)	5 (63%)
2 *n* (%)	10 (34%)	7 (33%)	3 (37%)
>2 *n* (%)	6 (21%)	6 (29%)	
Prior platinum therapy	28 (97%)	21 (100%)	7 (87%)

^a^Includes one patient each with renal and thymic primary.

^b^One patient with unknown differentiation.

### Outcomes and adverse events

**Fig. 1:**
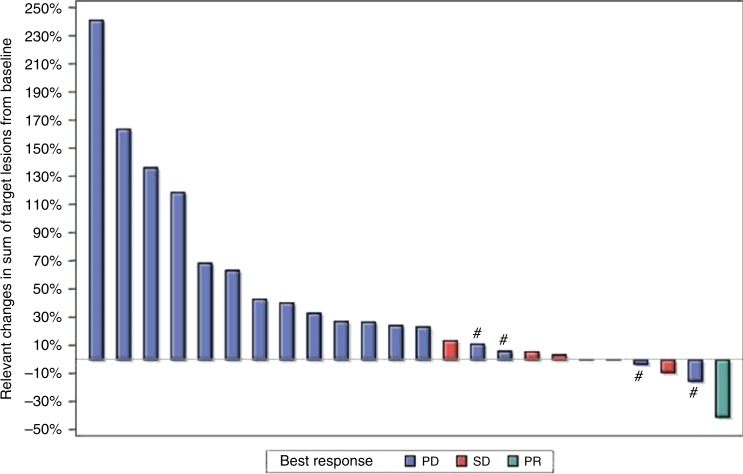
Waterfall plot depicting best overall response to therapy by patient. Five patients were not evaluable for response. # represents patients who progressed due to appearance of new lesions despite reduction or less than 20% increase in tumor size. PD progressive disease, SD stable disease, PR partial response.

**Fig. 2:**
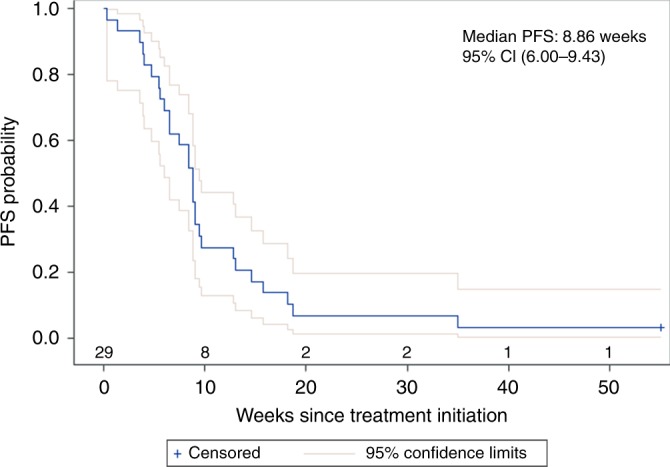
Kaplan-Meier curve showing progression free survival (PFS) among 29 patients who received Pembrolizumab. Median PFS 8.86 weeks (95% confidence interval 6.00–9.43).

**Fig. 3:**
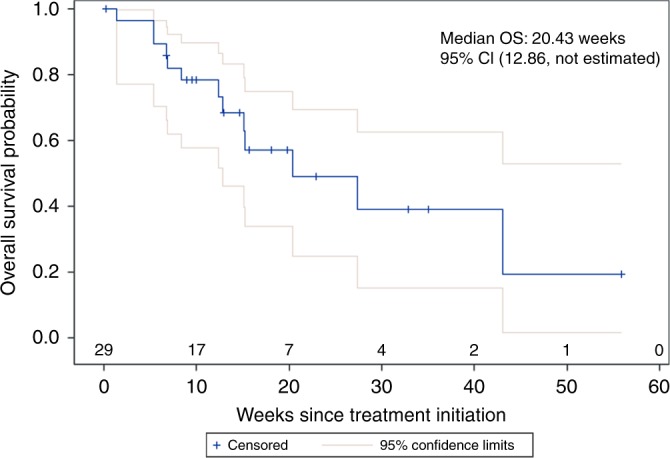
Kaplan Meier curve showing overall survival since treatment initiation among 29 patients who received Pembrolizumab. Medial overall survival was 20.43 weeks (95% confidence interval 12.86- not estimated).

Only one patient (3.4%, CI 0.1–17.8%) with a large cell oesophageal neuroendocrine carcinoma had an objective partial radiographic response (PR) that was ongoing for 13 months, at which time the patient decided to withdraw from the study. Six patients (20.7%, CI 7.9–39.7%) had stable disease (SD), resulting in a disease control rate (DCR) of 24.1% (PR + SD). Seventeen patients (58.6%, CI 38–9%–76.5%) experienced disease progression as their best response. Five patients (17%) were unevaluable due to clinical progression before first scheduled imaging. Efficacy outcomes are listed in Table 2 and a waterfall plot depicting objective radiographic responses is shown in Fig. 1. Patient demographic variables (gender, age, PS, differentiation, Ki-67 and prior therapies) did not affect DCR significantly (p > 0.05). Median PFS was 8.9 weeks and median OS was 20.4 weeks in the combined pool (Figs. 2, 3). Both PFS and OS were not affected by patient variables (*p* > 0.05). Two patients (besides the one responder) had a durable disease control lasting more than 18 weeks. Both were well-differentiated G3 NETs with Ki-67 in the 20–30% range, only one of whom had RECIST progression over the 3 months prior to study enrolment. The most common adverse events were fatigue and liver function abnormalities (Table 3)—findings that were similar to those in previous reports.^36,37^ Pembrolizumab was well tolerated with only 9 grade 3 events and no grade 4 events considered at least possibly drug-related.

**Table 2:** Response to Pembrolizumab therapy.

	Combined	FC	LM
Best overall response	*N* (%)	*N* (%)	*N* (%)
ORR (PR)	1 (3.4%)	1 (4.7%)	0
Stable disease	6 (20.7%)	4 (19.1%)	2 (25%)
Disease control rate (CR + PR + SD)	7 (24.1%)	5 (23.8%)	2 (25%)
Progressive disease	17 (58.6%)	12 (57.1%)	5 (62.5%)
Not evaluated	5 (17.2%)	4 (9.5%)	1 (12.5%)

**Table 3:** Adverse events.

Adverse event^a^	Grade			
	All	1	2	3
	*N*	*N*	*N*	*N*
Fatigue	7	3	4	.
Arthralgia	1	1	.	.
Oedema	2	2	.	.
Nausea/vomiting	3	3	.	.
Diarrhoea	2	1	1	.
Alkaline phosphatase increased	3	.	.	3
Aspartate aminotransferase increased	3	.	.	3
Other gastrointestinal (dry mouth, anorexia, bloating, rectal pain)	6	3	2	1
Hypercalcaemia	1	.	.	1
Hyperkalaemia	1	.	.	1
Hypothyroidism	1	.	1	.
Lymphocyte count decreased	2	.	2	.
Injection site reactions	1	1	.	.
Skin (rash, pruritis etc)	3	3	.	.
All	36	17	10	9

^a^Possible, probable and definitely related to study drug (Highest grade for each toxicity per patient).

### Correlative studies

Fifteen of 21 patients from the FC cohort had available archival tissue evaluated for PD-L1 staining. Seven tumour samples (47%) stained positive for PD-L1 (>1%) and three (20%) additional samples were positive for staining in the tumour-stromal interface. Eight tumour samples (53%) had evidence of TILs >2 + (>10 TILs/HPF). There was no difference in the disease control rate (DCR), PFS or OS between the PD-L1-negative and -positive groups (*p* 0.56, 0.88 and 0.55, respectively). The tumour sample from the patient with partial response to therapy did not stain positive for PD-L1 but had >20 TILs/HPF. This patient also had extensive lymphoplasmacytic peri-tumoural response seen on H&E slides with a reactive stromal interface.

## Discussion

Despite prior evidence of activity of CPI immunotherapy in neuroendocrine cancers originating in the lung and skin, limited evidence of activity with pembrolizumab was observed in this combined analysis of two studies of G3 NENs originating in other organs. We also found that despite a higher baseline PD-L1 expression (47% with positive staining), there was no correlation between PD-L1 expression and response to therapy. In hindsight, this is not surprising. SCLC is almost uniformly associated with a history of heavy tobacco use, whereas Merkel cell carcinoma occurs in the setting of extensive ultraviolet (UV) light exposure or Merkel cell polyoma virus. Both tobacco use and UV light exposure can confer an exceptionally high tumour mutational burden, leading to expression of large numbers of tumour neoantigens. Virus-positive Merkel cell carcinomas express viral oncoproteins which are also immunogenic. These environmental causes of immunogenicity are typically lacking in neuroendocrine cancers originating in the gastrointestinal tract, genitourinary tract and other sites. These accumulating data all indicate that, with rare exceptions, PD-1 inhibitor monotherapy is minimally active in NENs originating outside of the lung or skin, regardless of tumour grade or differentiation.

Our results corroborate findings from other studies of PD-1 inhibitors in NENs. A large phase 2 study of spartalizumab, a PD-1 inhibiting antibody enrolled 95 patients with low and intermediate-grade NENs (divided roughly equally among GI, pancreatic and lung primaries) and 21 patients with G3 NENs, demonstrating response rates of 7.4% in well-differentiated NENs, and only 4.8% in poorly differentiated carcinomas.^38^ In the well-differentiated NEN cohort of the Keynote 158 study, the objective response rate was only 3.7%.^39^

Further studies are needed to explain the underlying mechanisms of response and resistance, identify predictive markers of potential benefit, and determine the optimal immunotherapeutic combination in this disease setting. Preliminary data from a phase 2 basket study of ipilimumab and nivolumab in rare cancers suggest that a combination of CTLA-4 and PD-1 inhibition may yield a higher response rate than pembrolizumab monotherapy. In an unplanned subset analysis of G3 NENs, objective responses were observed in 8/19 patients (42%).^40^ These results require validation in larger prospective trials. Other studies in SCLC have validated the concept of adding immunotherapy to a chemotherapy backbone by demonstrating a statistically significant, albeit modest, improvement in OS.^30,41^ It is unclear whether this approach can be translated to extrapulmonary high-grade NENs.

We found a high baseline PD-L1 expression (47% with positive staining) in our patient cohort, but there was no correlation between PD-L1 expression and response to therapy. The one patient who responded to therapy in our cohort had a high number of TILs despite negative PD-L1 expression. Other groups have also reported such expression to be between 14% and 50%. In one study the expression of PD-L1 was not affected by the primary site of extrapulmonary G3 NEN but varied depending on tumour differentiation, with lower expression seen in well-differentiated NET G3.^42^

Our studies accrued patients rapidly (in less than 18 months), signalling that trials of high-grade NENs are not only possible but actively sought out by patients with this cancer, paving the way for more trials specific to this tumour type. Patients were enrolled at three comprehensive cancer centres with standardised pathology and radiology review processes, crucial for appropriate classification of these tumours. The limitations of our study include the non-randomised single-arm trial design, a small sample size, and the inclusion of a heterogeneous patient population with both well and poorly differentiated histology. However, the primary objective of our study was evaluation of response rate, an endpoint that may be less affected by patient heterogeneity than PFS.

## Conclusions

Pembrolizumab monotherapy is minimally active in high-grade NENs progressing after at least one prior line of therapy. Novel treatment strategies, including potential immunotherapy-based combinations, will need to be investigated in order to improve the poor prognosis currently associated with high-grade NENs.

## Data Availability

The data are available for all study authors. The datasets used and analysed during the current study are available from the corresponding author on reasonable request.
